# Inter-Group Face Recognition Bias Was Modulated by the Group Status

**DOI:** 10.3389/fpsyg.2022.837836

**Published:** 2022-05-27

**Authors:** Bingjie Hu, Linlin Yan, Chengyan Zheng, Yuhao Tang, Qiuye Lin, Wenling Xia, Zhe Wang

**Affiliations:** Department of Psychology, Zhejiang Sci-Tech University, Hangzhou, China

**Keywords:** own-group face recognition bias, in-group members, out-group members, competitive cues, group status, in-group identification

## Abstract

Previous studies have shown that social categorization can induce an own-group face recognition bias. However, similar and better other-group face recognition emerged recently. In this research, we aimed to examine whether competitive cues and group status accompanied by social categorization can modulate the inter-group face recognition bias. Moreover, we investigated how the group identification of individuals with different statuses affected the inter-group face recognition bias. The results indicated that an own-group face recognition bias emerged for targets with in-group labels compared to out-group labels. Moreover, when the group labels signaled competitive cues, the own-group face recognition bias was reversed. Furthermore, low-status and similar-status individuals exhibited out-group face recognition bias, but high-status individuals did not. In addition, the higher the in-group identification scores of participants from the low-status group, the stronger the out-group face recognition bias. These results suggested that competitive cues would reverse the own-group face recognition bias and the group status would play a modulating role in face recognition bias.

## Introduction

People are better at recognizing the faces of their in-group members than that of out-group members, which is termed as the own-group memory bias (OGB, Bernstein et al., 2007). The bias in face recognition is surprisingly robust across a wide variety of categories, such as race (Meissner and Brigham, 2001), gender (Herlitz and Lovén, 2013; Wolff et al., 2014), sexual orientation (Rule et al., 2007), religion (Rule et al., 2010), political party (Ray et al., 2010), and university affiliation (Bernstein et al., 2007).

A majority of evidence (Hugenberg et al., 2011) showed that social categorization into in-group and out-group was sufficient to elicit the OGB, but a series of studies (Shriver et al., 2008; Van Bavel et al., 2008, 2011; Ng et al., 2016, 2020; Yan et al., 2017; Harrison et al., 2020; Fuller et al., 2021) have continued to report inconsistent results, especially when there were no salient physiognomic features on faces (same-race faces without other categorical diagnostic features). For example, some studies showed equivalent recognition memory for in-group versus out-group faces (Yan et al., 2017 for Chinese populations; Ng et al., 2016, 2020 for first-generation East Asian Canadians; Harrison et al., 2020 and Fuller et al., 2021 for United Kingdom populations). In-/out-groups categorized by university membership which was a lack of competitive or rivalrous relationships did not elicit an own-group memory bias (Harrison et al., 2020; Fuller et al., 2021). Furthermore, some studies even showed that out-group faces were better recognized than in-group faces when participants were in a competitive situation that required attention allocation toward out-group members (Shriver and Hugenberg, 2010, the White with an other-race recognition bias due to the Black with high-status occupational titles; Van Bavel and Cunningham, 2012, the observers with an out-group recognition bias due to their “Spy” role; Fuller et al., 2021, the football fans with an out-group recognition bias due to the threat from out-group members). Therefore, it could be speculated that some competitive cues underlying the group labels and not simple categorization elicited the inter-group memory bias.

More than that, social categorization with the same group labels signaled different competitive relationships between groups to observers. For example, Snibber et al. (2003) found that some participants preferred the out-group members from universities with better academic reputations rather than preferred in-group members from the university with better sports reputations. However, some participants from both universities evaluated their in-group members more positively than out-group members regardless of the reputation types of the universities. And the competitive relationship signaled by group labels was not limited to academic or sports reputation, but geographical distance and physical proximity to each other (Van Bavel and Cunningham, 2012; Ng et al., 2016; Xiao et al., 2016; Yan et al., 2017). Since competitive opponents represent a relevant source of information or threat within the social environment, observers would modulate the gazing behavior, attention resource, and memory (Kilduff et al., 2010; Ciardo et al., 2015; Liu et al., 2020). There is reason to doubt that only perceived competitive (or rivalry) relationships may play an important role in modulating an inter-group recognition bias.

As a result, the competition will lead to status differentials between groups, and the individuals with different statuses may process their in-group and out-group faces in different ways. For example, most studies (Meissner and Brigham, 2001) demonstrated an own-race recognition bias (i.e., a typical own-group bias) in the Blacks and Whites. However, Wright et al. (2003) found that Blacks living in South Africa showed better recognition for Whites (out-group members) compared to Blacks (in-group members), but Whites both in South Africa and in England showed better recognition for Whites (in-group members) compared to Blacks (out-group members). They speculated that the out-group recognition bias from the Blacks was due to the Whites in South Africa are in power. Considering that information regarding the social status was extracted from faces rapidly (Dalmaso et al., 2014), target status might play an important role in face processing from attention to feature integration (Ratcliff et al., 2011; Dalmaso et al., 2012). However, there were no more direct studies to examine how the relative status of individuals affects the face recognition performance of in-group and out-group members. In addition, as individuals hold multiple social identities, in-group identification would influence the inter-group recognition bias. As an individual’s salient identity shifts, an out-group member in one situation may be recategorized as an in-group in another (Hehman et al., 2010). More than that, selective attention would be guided toward motivationally relevant social groups (Park et al., 2016). Considering the social identity of individuals would shape social attention and memory (Van Bavel and Cunningham, 2012), and it was necessary to investigate how in-group identification modulates the inter-group bias in face recognition of the individuals with different statuses.

Given that competitive cues signaled by group labels will motivate individuals belonging to different-status groups to perceive and recognize others differently, we speculated that social categorization manipulated with competitive information and group status may modulate the inter-group face memory bias. In this research, we aimed to replicate and extend the findings of Bernstein et al. (2007) by exploring the effect of competitive cues (Experiment 1) and group status (Experiment 2) signaled by group membership on the inter-group face memory bias. Specifically, we chose the uniform colors and logos as group membership and group status to explore how competitive cues and group status modulate the inter-group face recognition bias. If competitive cues play a role in the inter-group face recognition bias, then participants with or without the knowledge of competitive information from group membership will show different patterns of recognition performance for in-group and out-group members. In particular, without the knowledge of competitive information from group membership, in-group members are recognized better than out-group members (i.e., own-group face recognition advantage). And with the knowledge of competitive information from group membership, out-group members are recognized as equivalent to or better than in-group members (i.e., own-group face recognition advantage decreasing or reversing). If group status affects the inter-group face recognition bias, then participants belonging to and identified with different-status groups will show different patterns of inter-group face recognition bias. Namely, the observers assigned to the low-status and the same-status group will show the reversed own-group face recognition advantage (i.e., better recognition for out-group members than in-group members), especially for the individuals with stronger in-group identification. While the high-status group members will remain the own-group face recognition advantage (i.e., better recognition for in-group members than out-group members), especially for the individuals with stronger in-group identification.

## Experiment 1 Group Membership With/Without Competitive Relationship

### Methods

#### Participants and Design

A total of 64 Chinese undergraduates (25 males, mean age = 19.9 years, *SD* = 1.7) from Zhejiang Sci-Tech University took part in the study, separated randomly into one group with competitive cues and the other group without competitive cues. The group without competitive cues consisted of 32 participants, half of them were separated randomly into learning faces with a red uniform as in-group members and half of them into learning faces with a green uniform as in-group members. The group with competitive cues consisted of 32 participants, half of them were separated randomly into a LION team and half of them into a SHEEP team. The LION team consisted of 16 participants who were learning faces with red uniform attached LION logo as in-group members and faces with green uniform attached SHEEP logo as out-group members. The SHEEP team consisted of 16 participants that were learning faces with red uniforms attached SHEEP logo as in-group members and faces with green uniforms attached to the LION logo as out-group members. The experiment had a 2 (Group membership: in-group vs. out-group) × 2 (Instruction cues: competitive vs. non-competitive) mixed design, with the last factor between subjects. All participants received payment for participating in the experiment and reported normal or corrected-to-normal vision. The experiment was approved by the Human Research Ethics Committee of Zhejiang Sci-Tech University, and all participants gave informed consent to participate in the study. The sample size of the current experiment was determined from two considerations. First, we calculated the sample sizes (ranging from 26 to 37 for each group) of some published studies (Shriver et al., 2008; Hehman et al., 2010) that used the same old/new paradigm with the power value of 0.8. Second, the ideal sample size (34 for each group) was estimated by using G-power 3.1^1^ with a power value of 0.8 and a medium effect size of 0.5 (Cohen, 1988) when α = 0.05. The estimated sample sizes were similar to the actual sample size in this experiment. The sample sizes of the following experiments were based on the same criteria.

#### Stimuli

Totally 32 Chinese male hairless full-front faces from the face pool of Kang Lee’s lab were used as the stimuli, unfamiliar to the participants, posing with a neutral expression. Adobe Photoshop was used to edit the images to get rid of specific features and resize them to approximately 16.9 cm × 22.2 cm (including face and upper body), all faces have the same outline located at the center of the screen. The 32 faces were presented separately with a red team uniform, a green team uniform, or a blue team uniform against gray background. There were Lion and Sheep logos attached at the upper left corner of the uniforms indicating specific teams, respectively, in the learning phase and recognition phase of the competitive condition.

#### Procedure

**FIGURE 1:**
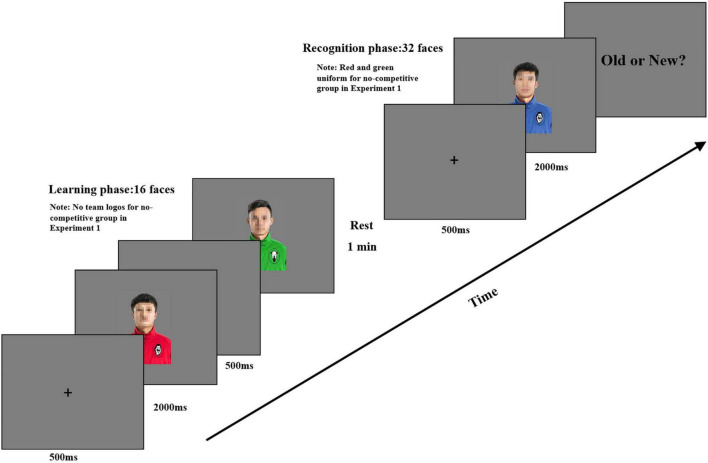
The procedure for competitive group in Experiment 1.

After providing informed consent, all participants were asked to complete an old/new face recognition task consisting of a learning phase and a recognition phase (Figure 1). All instructions and stimuli were presented with E-Prime 2.0 (Psychology Software Testing, Pittsburgh, PA, United States) *via* a computer. At the same time, all behavioral data would be collected by E-Prime 2.0. During the face recognition task, the participants were instructed that they would see some faces on the computer screen and should attend closely to these faces to recognize them later. The participants from the no-competitive group were asked to wear either a red or a green wristband corresponding to their team uniforms and were reminded that the wristband identified them as a member of their group. Different from the no-competitive group, the participants from the competitive group were instructed on the guidelines: “There are two baseball teams, one is the Lion team, one is the Sheep team. The Lion team has the same status as the Sheep team. You belong to Lion (or Sheep) team.” Then each trial started with a 500-ms fixation cross, followed by a face wearing a red or green uniform tagged with the team logo Lion or Sheep for 2,000 ms, and the interstimulus interval is 500 ms. After learning 16 different faces with specific uniforms, participants had a 1-min rest. Then participants were instructed that they would see a series of faces wearing a blue uniform with specific team logos, some of which they had seen (i.e., old faces) during the learning phase and some of which they had not seen before (i.e., new faces). Participants were instructed to decide as accurately as possible whether the target face was seen or not (left or right keys, counterbalanced across participants) with a maximum display time of 2,000 ms. There was an unlimited response time unless participants responded to the target face. All the 32 faces were presented in random order during the recognition phase including the 16 faces previously seen during the learning phase and 16 new faces with red or green team uniforms in the no-competitive group. But 16 old faces and 16 new faces with blue uniforms with Lion or Sheep team logos were presented in the competitive group.

### Results

Of interest was the extent to which the group membership influenced face recognition. Thus, measures of sensitivity (*d’* = z [Hits]-z [FA]) and response bias (*C* = −0.5 [z (Hits) + z (FA)]) were computed according to the signal detection theory (Green and Swets, 1966), which can be created from hit rates (the correct identification of an old face) and false-alarms rates (the misidentification of a new face as an old face) separately for in-group targets and out-group targets. When hit rates and false-alarm rates were 100% or 0%, a standard correction “1-1/2N” and “1/2N” was replaced separately, where N is the maximum number of targets (Macmillan and Kaplan, 1985).

#### Recognition Accuracy (*d’*)

**FIGURE 2:**
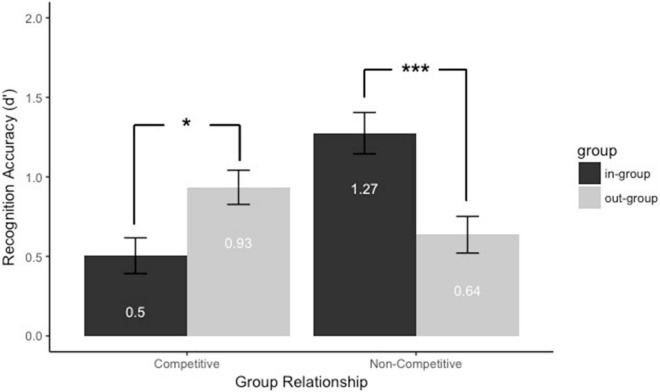
Recognition accuracy (d’) for faces assigned to the different groups with and without competitive cues. Black bars refer to in-group members, and gray bars refer to out-group members. Error bars represent standard errors (*p < 0.05 and ***p < 0.001).

To test whether the presence of group membership and group relationship influenced face recognition, we conducted a 2 (Group membership: in-group vs. out-group) × 2 (Group relationship: competitive vs. non-competitive) mixed-model analysis of variance. The results (Figure 2) showed no main effect of group membership [*F*(1,62) = 0.93, *p* = 0.34, *η_*p*_^2^* = 0.015]. There was a marginally significant main effect of group relationship [*F*(1,62) = 3.58, *p* = 0.063, *η_*p*_^2^* = 0.055]. Participants assigned to the no-competitive group (*M* = 0.96, *SE* = 0.09) recognized faces marginally better than those assigned to the competitive group (*M* = 0.72, *SE* = 0.09). However, the interaction between group membership and instruction cues was significant, *F*(1,62) = 24.52, *p* < 0.001, *η_*p*_^2^* = 0.28. Then we did the follow-up comparison between group membership and group relationship. First, we explored the type of the group membership would affect the face recognition when assigned to the groups with different group relationship. When participants were assigned to the competitive group, faces as the in-group members (*M*_*in–group*_ = 0.5, *SE*_*in–group*_ = 0.12) were worse recognized than were faces as the out-group members (*M*_*out–group*_ = 0.93, *SE*_*out–group*_ = 0.11), *p* = 0.012, *d* = 0.47. When participants were assigned to the no-competition group, faces as the in-group members (*M*_*in–group*_ = 1.27, *SE*_*in–group*_ = 0.12) were better recognized than were faces as the out-group members (*M*_*out–group*_ = 0.64, *SE*_*out–group*_ = 0.11), *p* < 0.001, *d* = 0.77. Second, we compared competitive and non-competitive groups faces as in-group and out-group members separately. Faces as the in-group members were better recognized when participants were assigned to the non-competitive group than the competitive group, *p* < 0.001, *d* = 1.11. Faces as the out-group members were marginally better recognized when participants were assigned to the competitive group than the non-competitive group, *p* = 0.063, *d* = 0.46. Therefore, these results suggested that the overall face recognition performance was not improved but the inter-group face recognition bias was modulated by the group relationship. Individuals in the competitive group showed out-group face recognition bias, but individuals in the no-competitive group showed own-group face recognition bias.

#### Response Bias (C)

**FIGURE 3:**
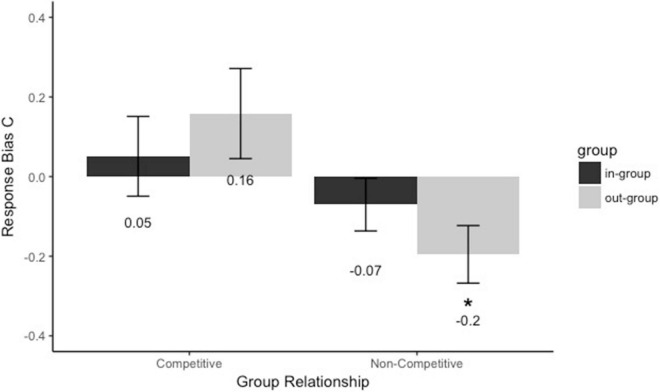
Response bias C for faces assigned to the different groups with and without competitive cues. Black bars refer to in-group members, and gray bars refer to out-group members. Error bars represent standard errors (*p < 0.05).

To test whether the presence of group membership and group relationship influenced response bias, we conducted a 2 (Group membership: in-group vs. out-group) × 2 (Group relationship: competitive vs. non-competitive) mixed-model analysis of variance. The results (Figure 3) showed no main effect of group membership [*F*(1,62) = 0.02, *p* = 0.89, *η_*p*_^2^* < 0.001] and a significant main effect of group relationship [*F*(1,62) = 4.57, *p* = 0.037, *η_*p*_^2^* = 0.069]. Participants assigned to the competitive group (*M* = 0.11, *SE* = 0.08) were stricter to recognize faces than those assigned to the non-competitive group (*M* = −0.13, *SE* = 0.08). And the interaction between group membership and group relationship was marginally significant, *F*(1,62) = 3. 48, *p* = 0.067, *η_*p*_^2^* = 0.053. Then, we did the follow-up comparison between group membership and group relationship. First, we explored whether the type of the group membership would affect the response bias when assigned to the groups with different group relationship. Participants assigned to the competitive group were stricter to recognize out-group members (*M*_*competitive–group*_ = 0.16, *SE*_*competitive–group*_ = 0.11) than those assigned to the non-competitive group (*M*_*non–competitive–group*_ = −0.2, *SE*_*non–competitive–group*_ = 0.07), *p* = 0.01, *d* = 0.67. But there was no difference to recognize in-group members when participants were assigned to the competitive group (*M*_*competitive–group*_ = 0.05, *SE*_*competitive–group*_ = 0.1) or the non-competitive group (*M*_*non–competitive–group*_ = −0.07, *SE*_*non–competitive–group*_ = 0.07), *p* = 0.32, *d* = 0.25. Second, we compared response bias C between competitive and non-competitive groups in faces as in-group and out-group members separately. Faces as the out-group members were more strictly recognized when participants were assigned to the competitive group than the non-competitive group, *p* = 0.011, *d* = 0.67. But there was no response bias between faces as the in-group members from the competitive group and non-competitive group, *p* = 0.32, *d* = 0.25. Moreover, the response bias *C* of the participants assigned to the non-competitive group was more relaxed to say “Yes” to the out-group faces (*C* = −0.2 < 0, *p* = 0.01). The results suggested that the response bias of the participants was modulated by the group relationship during the out-group face recognition. Individuals in the competitive group were stricter to recognize out-group members than individuals in the non-competitive group, but no response bias in recognizing in-group members between individuals in the competitive group and those in the non-competitive group.

## Experiment 2 Competitive Relationship With Different Status

### Methods

#### Participants and Design

A total of 96 Chinese undergraduates (30 males, mean age = 19.8 years, *SD* = 1.3) from Zhejiang Sci-Tech University took part in the study, separated randomly into a different-status group (team logos attached to uniforms indicated as status, Lion as high status and Sheep as low status) and same-status group (Lion and Sheep as the same status). The different-status group consisted of 64 participants, half of them were separated randomly into a high-status team and half of them into a low-status team. The high-status team consisted of 32 participants that were learning faces with red uniform attached LION logo as in-group members and learning faces with green uniform attached SHEEP logo as out-group members. The low-status team consisted of 32 participants that were learning faces with red uniform attached SHEEP logo as in-group members and learning faces with green uniform attached LION logo as out-group members. However, 30 participants were left in the final data analysis of the low-status team, as two participants were removed for incorrectly identifying which team they were assigned to. The same-status group consisted of 32 participants with the same assignment as the competitive group in Experiment 1. The experiment had a 2 (Group membership: in-group vs. out-group) × 3 (Group Status: same, high vs. low) mixed design, with the last factor between subjects. All participants received payment for participating in the experiment and reported normal or corrected-to-normal vision. The experiment was approved by the Human Research Ethics Committee of Zhejiang Sci-Tech University, and all participants gave informed consent to participate in the study.

#### Stimuli

The faces used in Experiment 2 were as same as those in Experiment 1. There were Lion and Sheep logos attached at the upper left corner of the uniforms indicating different teams, respectively, in the learning phase and recognition phase.

A three-item in-group identification scale according to social identity literature (Ashmore et al., 2004) was used to measure self-identification with the assigned in-group membership. Three items from this scale were as follows: “I’m proud to be a member of the Lion (Sheep) group,” “It’s important for me to be a member of the Lion (Sheep) group,” and “I value being a member of the Lion (Sheep) group.” The responses were given on a five-point Likert-type scale from 1 (strongly disagree) to 5 (strongly agree). The higher the score, the more highly identified with assigned in-group membership.

#### Procedure

After providing informed consent, all participants were asked to complete an old/new face recognition task. All instructions and stimuli were presented with E-Prime 2.0 *via* a computer. At the same time, all behavioral data would be collected by E-Prime 2.0. During the face recognition task, participants assigned to the group with high status or low status were instructed on the guidelines: “There are two baseball teams, one is the Lion team, one is the Sheep team. The Lion team is higher than the Sheep team. You belong to Lion (or Sheep) team.” Participants assigned to the group with the same status were instructed as the competitive group in Experiment 1. Then each trial started with a 500-ms fixation cross, followed by a face wearing a uniform tagged with the team logo Lion or Sheep for 2,000 ms, and the interstimulus interval is 500 ms. After learning 16 different faces with specified uniforms, participants had a 1-min rest. Then, participants were instructed that they would see a series of faces wearing a blue uniform with specific team logos, some of which they had seen (i.e., old faces) during the learning phase and some of which they had not seen before (i.e., new faces). Participants were instructed to decide as accurately as possible whether the target face was seen or not (left or right keys, counterbalanced across participants) with a maximum display time of 2,000 ms. There was an unlimited display time unless participants responded to the target face. All the 32 faces were presented in random order during the recognition phase including the 16 faces previously seen during the learning phase and 16 new faces with blue uniforms with the Lion or Sheep team logo separately. After participants completed face recognition, they completed an in-group identification scale.

### Results

#### Recognition Accuracy (*d’*)

**FIGURE 4:**
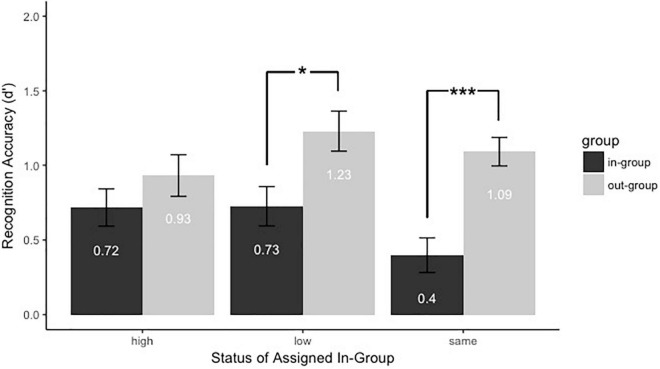
Recognition accuracy (d’) for faces assigned to different groups with high, low, or same power. Black bars refer to in-group members, and gray bars refer to out-group members. Error bars represent standard errors (*p < 0.05 and ***p < 0.001).

To test whether the presence of group membership and group status influenced face recognition, we conducted a 2 (Group membership: in-group vs. out-group) × 3 (Group Status: same, high vs. low) mixed-model analysis of variance. There was a significant main effect of group membership [*F*(1,91) = 27.59, *p* < 0.001, *η_*p*_^2^* = 0.23]. Faces assigned to the out-group (*M* = 1.08, *SE* = 0.07) were recognized better than those assigned to the in-group (*M* = 0.61, *SE* = 0.07). The results (Figure 4) indicated that most participants had superior memory for out-group members. There was no main effect of group status [*F*(2,91) = 1.46, *p* = 0.24, *η_*p*_^2^* = 0.031]. However, the interaction between group membership and group status was marginally significant, *F*(2,91) = 2.49, *p* = 0.089, *η_*p*_^2^* = 0.052. Then, we did a follow-up comparison between group membership and group status. First, we explored the type of the group membership would affect the face recognition when assigned to the groups with different statuses. When participants assigned to the group with same status, faces as the in-group members (*M*_*in–group*_ = 0.4, *SE*_*in–group*_ = 0.12) were worse recognized than faces as the out-group members (*M*_*out–group*_ = 1.09, *SE*_*out–group*_ = 0.1), *p* < 0.001, *d* = 0.91. When participants assigned to the low-status group, faces as the in-group members (*M*_*in–group*_ = 0.73, *SE*_*in–group*_ = 0.13) were worse recognized than faces as the out-group members (*M*_*out–group*_ = 1.23, *SE*_*out–group*_ = 0.13), *p* = 0.011, *d* = 0.49. However, different from what we expected, when participants assigned to the high-status group, there was no significant difference between faces (*M*_*in–group*_ = 0.72, *SE*_*in–group*_ = 0.12) as in-group members and out-group members (*M*_*out–group*_ = 0.93, *SE*_*out–group*_ = 0.14), *p* = 0.15, *d* = 0.26. Second, we compared different group statuses in faces as in-group and out-group members separately. Faces as in-group members were marginally better recognized when participants were assigned to the high-status group (High vs. Same, *p* = 0.068) and the low-status group (Low vs. Same, *p* = 0.066) than the group with the same status. In addition, faces as out-group members were marginally better recognized when participants were assigned to the low-status group than the high-status group (Low vs. High, *p* = 0.095), and there were no other significant effects (*p*s > 0.05). These results suggested that the overall face recognition performance was not influenced but the inter-group face recognition bias was modulated by the group status. As predicted, individuals in the low-status and same-status groups showed reversed own-group recognition advantage. But individuals in the high-status group showed equivalent recognition performance between in-group and out-group members.

#### Response Bias (*C*)

**FIGURE 5:**
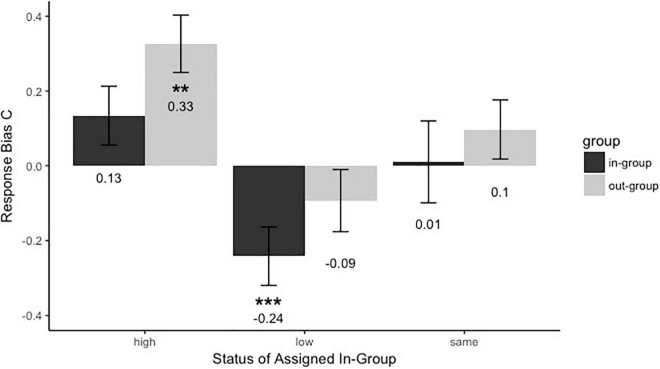
Response bias C for faces assigned to different groups with high, low, or same power. Black bars refer to in-group members, and gray bars refer to out-group members. Error bars represent standard errors (**p < 0.01 and ***p < 0.001).

To test whether the presence of group membership and group status influenced response bias, we conducted a 2 (Group membership: in-group vs. out-group) × 3 (Group Status: same, high vs. low) mixed-model analysis of variance. The results (Figure 5) showed a significant main effect of group membership [*F*(1,91) = 6.44, *p* = 0.013, *η_*p*_^2^* = 0.066]. The out-group faces (*M*_*out–group*_ = 0.11, *SE*_*out–group*_ = 0.046) were more strictly recognized than those in-group faces (*M*_*in–group*_ = −0.032, *SE*_*in–group*_ = 0.052). And there was a significant main effect of group status [*F*(2,91) = 7.95, *p* = 0.001, *η_*p*_^2^* = 0.15]. Participants assigned to the low-status group (*M*_*low–status*_ = −0.17, *SE*_*low–status*_ = 0.072) were more relaxed to say “have seen” than those assigned to the same group (*M*_*same–group*_ = 0.054, *SE*_*same–group*_ = 0.069, Low vs. Same, *p* = 0.029) and the high-status group (*M*_*high–status*_ = 0.23, *SE*_*high–status*_ = 0.069, Low vs. High, *p* < 0.001). And participants assigned to the same group were marginally more relaxed to respond “have seen” than those assigned to the high-status group (Same vs. High, *p* = 0.075). However, the interaction between group membership and group status was not significant, *F*(2,91) = 0.3, *p* = 0.74, *η_*p*_^2^* = 0.007. Then, we explored how the group status would affect the response bias during face recognition. Participants assigned to the high-status group were stricter to say “have seen” to the out-group members (*C* = 0.33 > 0, *p* = 0.004). Participants assigned to the low group were more relaxed to say “have seen” to the in-group members (*C* = −0.24 < 0, *p* < 0.001). The other response bias *C*s were no different from zero (*ps* > 0.05). The results suggested that the response bias of the participants was modulated by the group membership and group status during the face recognition. Individuals tended to recognize out-group members with a stricter strategy, and those in high-status and same-status groups were stricter to recognize members than individuals in the low-status group.

#### Relationship Between In-Group Identification and Face Recognition Bias

**FIGURE 6:**
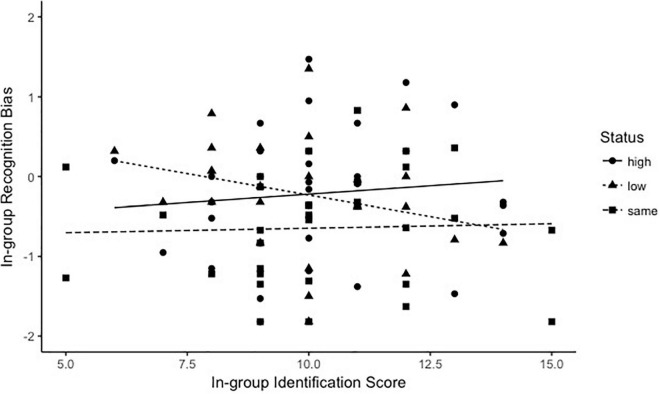
The correlation between the in-group identification (*x*-axis) and the in-group face recognition bias in d’ (*y*-axis). The solid line refers to the group with the same power; the dotted line refers to the high group; and the dashed line refers to the low group.

To examine the relationship between in-group identification and face recognition bias between in-group and out-group members, we first computed the size of the own-group recognition advantage in discriminability *d’* by subtracting each participant’s *d’* for recognizing the in-group faces from that for recognizing the out-group faces. And then a correlation analysis was carried out across the participant between the in-group identification scores and own-group recognition advantage in discriminability *d’*. There was a significant negative correlation between the in-group identification score and in-group face recognition bias when the participants were assigned to the low-status group (see Figure 6; *r* = −0.37, *p* = 0.043). However, there was no significant correlation between the in-group identification score and in-group face recognition bias when the participants were assigned to the high-status group (*r* = 0.1, *p* = 0.58) or the group with same status (*r* = 0.039, *p* = 0.83). And there were no significant correlations between in-group identification scores and in-group face recognition criterion (*ps* > 0.05). The results suggested that the higher the in-group identification scores of participants from the low-status group, the lower the in-group face recognition bias.

## General Discussion

In this study, we replicated own-group face recognition bias using team membership as the experimentally manipulated social group. However, when there is a competitive relationship between groups, the overall recognition performance was not improved but the own-group face recognition bias was reversed. That is, individuals had better recognition of the faces of out-group members than that of in-group members. In addition, the status difference between groups would modulate the inter-group face recognition bias. Participants from the group with the same or low status showed out-group face recognition bias with a stricter strategy, but those from the group with high status did not. More than that, those who identified more highly with their in-group think of themselves in the low-status group and showed worse own-group face recognition bias.

In Study 1, we found that competitive cues in instructions may motivate participants to recognize out-group members’ faces better than in-group members. Therefore, the own-group face recognition bias reversed. This finding contradicts previous studies showing that mere categorization by university affiliation is enough to bring about an own-group bias (OGB) in face recognition (Bernstein et al., 2007; Yan et al., 2017). Competitive opponents may represent a relevant threat to keep participants’ eyes on out-group members to improve face recognition. For example, competitive social interaction allocated more attentional resources to individuals with greater social relevance (Ciardo et al., 2015). Therefore, perceived rivalry from the out-group was a powerful psychological phenomenon that would affect team members’ motivation and performance (Kilduff et al., 2010). More than that, Harrison et al. found that the opposing or rivalrous cues between groups may play different roles in the recognition of in-/out-group members for two groups confronted with the different vote outcomes. In their study, an OGB was found for Remain supporters, but no OGB was found for Leave voters. Thus, it may be that the same competitive cues were likely to elicit different motivations in participants with different statuses to out-group members.

In Study 2, we found that the same competition cues were different for the group members with different statuses. Participants from the group with equal or low status recognized out-group members better than in-group members which replicated our findings in Study 1, while participants from the high-status group showed equal recognition performance of in-group and out-group members. Consistent with the out-group memory advantages for high-status targets demonstrated in the previous studies (Shriver and Hugenberg, 2010; Ratcliff et al., 2011), out-group members as powerful would be motivated to receive more face-processing resources or individuated styles of processing. Furthermore, solid evidence showed that eye gaze (Cheng et al., 2013) was preferentially focused on high-status individuals as well as covert attention (Jones et al., 2010; Dalmaso et al., 2012). That’s why participants from the group with equal or low status showed out-group recognition bias. Unlike perceivers with equal or low status, perceivers with high status recognize in-group members the same as out-group. Given that people selectively attend to the physical features of others to serve their goals (Maner et al., 2008; Sacco et al., 2009), it could be unnecessary for the superiors to allocate attention resources toward and memory for the lowers’ faces. More than that, Roberts et al. (2019) found that participants allocated more attention to low-status (low-dominance) relative to high-status (high dominance) faces, and no attention bias between low-status (low prestige) and high-status (high prestige) faces in the face of competing cognitive demands. More direct evidence (Fuller et al., 2021) also showed that perceivers with high threat recognized in-group members the same as out-group members. In addition, the correlations between participants’ identification with their in-group and inter-group recognition bias only emerged in low-status individuals and not in high-status individuals. That might be one of the reasons why high-status individuals did not show their own-group recognition advantage. Unlike the high-status individuals, we found that for the low-status individuals, the stronger in-group identification they were, the more the out-group face recognition bias. That might be because individuals with stronger in-group identification are more likely to extract facial cues and efficient encoding when out-group with high threat (Van Bavel and Cunningham, 2012; Hackel et al., 2014).

This study employed a minimal group procedure, which is useful in investigating perceivers’ intergroup face recognition biases without prior experience. Nonetheless, the generalizability of the findings to real-world context is questionable. Considerable evidence (Ackerman et al., 2006; Trawalter et al., 2008; Wilson et al., 2014; Yan et al., 2017; Harrison et al., 2020; Fuller et al., 2021) showed that the OGB can be moderated by out-group threat, different characteristics of the social groups and observers, and group salience. Moreover, some studies (Li and Yang, 2013; Chen et al., 2015) have reported that self-esteem could modulate attentional resource allocation of individuals and one’s self-esteem might be another potentially modulating factor in the inter-group face recognition bias. Therefore, a systematic investigation to bring all these factors to explore the origin of the OGB.

In conclusion, competitive cues would motivate perceivers to recognize better for others and the group status would play a modulating role in inter-group recognition bias. The flexible and appropriate evaluations of personal relationships with others are important for adaptive human behavior in a complex and dynamic social world.

## Data Availability Statement

The raw data supporting the conclusions of this article will be made available by the authors, without undue reservation.

## Ethics Statement

The studies involving human participants were reviewed and approved by the Human Research Ethics Committee of Zhejiang Sci-Tech University. The patients/participants provided their written informed consent to participate in this study. Written informed consent was obtained from the individual(s) for the publication of any potentially identifiable images or data included in this article.

## Author Contributions

LY and BH contributed to conception and design of the study. YT, QL, and WX organized the database and performed the statistical analysis. LY, BH, CZ, and ZW wrote the first draft of the manuscript. All authors contributed to manuscript revision, read, and approved the submitted version.

## Conflict of Interest

The authors declare that the research was conducted in the absence of any commercial or financial relationships that could be construed as a potential conflict of interest.

## Publisher’s Note

All claims expressed in this article are solely those of the authors and do not necessarily represent those of their affiliated organizations, or those of the publisher, the editors and the reviewers. Any product that may be evaluated in this article, or claim that may be made by its manufacturer, is not guaranteed or endorsed by the publisher.

## Footnotes

1
http://www.gpower.hhu.de/
